# Insights from multi-omics on GLP-1 receptor agonists in type 2 diabetes and obesity

**DOI:** 10.3389/fphar.2026.1812193

**Published:** 2026-10-13

**Authors:** Asma A. Elashi, Laila Hedaya, Viviana Maggio, Khaled Naja, Manfredi Rizzo, Mohamed A. Elrayess

**Affiliations:** 1 Biomedical Research Center, QU Health, Qatar University, Doha, Qatar; 2 School of Medicine, Promise Department, University of Palermo, Palermo, Italy; 3 Department of Medicine, Ras Al Khaimah (RAK) Medical and Health Sciences University, Ras AlKhaimah, United Arab Emirates; 4 College of Medicine, QU Health, Qatar University, Doha, Qatar

**Keywords:** diabetes, GLP-1, glucagon-like peptide-1, GLP-1RAs, metabolomics, microbiomics, omics, pharmacogenomics

## Abstract

Glucagon like peptide-1 receptor agonists (GLP-1RAs) have revolutionized the management of T2D and obesity. T2D clustering highlights distinct phenotypes that differ in GLP-1RA benefit. While they have markedly improved quality of life for many individuals, converging evidence indicates that genetic variation, metabolomic remodeling, and gut microbiota composition contribute to substantial inter-individual variability in GLP-1RA efficacy and tolerability. GLP-1RAs, in return, can variably affect the metabolome and microbiome composition in treated individuals. This review focuses on human multi-omics GLP-1RA intervention studies, complemented by relevant mechanistic preclinical evidence. Genetic variation in *GLP1R, TCF7L2*, and other loci has been associated with differences in glycaemic and weight outcomes, although most findings remain preliminary, inconsistent across populations, or not yet replicated. Pharmacometabolomic studies show recurrent of lipid-related pathways including, glycerophospholipids, sphingolipids, ceramides, amino acids, and bile acids, with drug-specific signatures. GLP-1RAs are also associated with changes in gut microbial composition, including taxa such as *Akkermansia* and *Bacteroides*, although the direction and clinical significance of these changes vary across studies due to methodological and population heterogeneity. Multi-omics and AI-based models show promise for predicting treatment response by integrating genetic, metabolic, microbial, and clinical data to supporting more effective and individualized therapy and advancing precision medicine.

## Graphical Abstract

**Figure:**
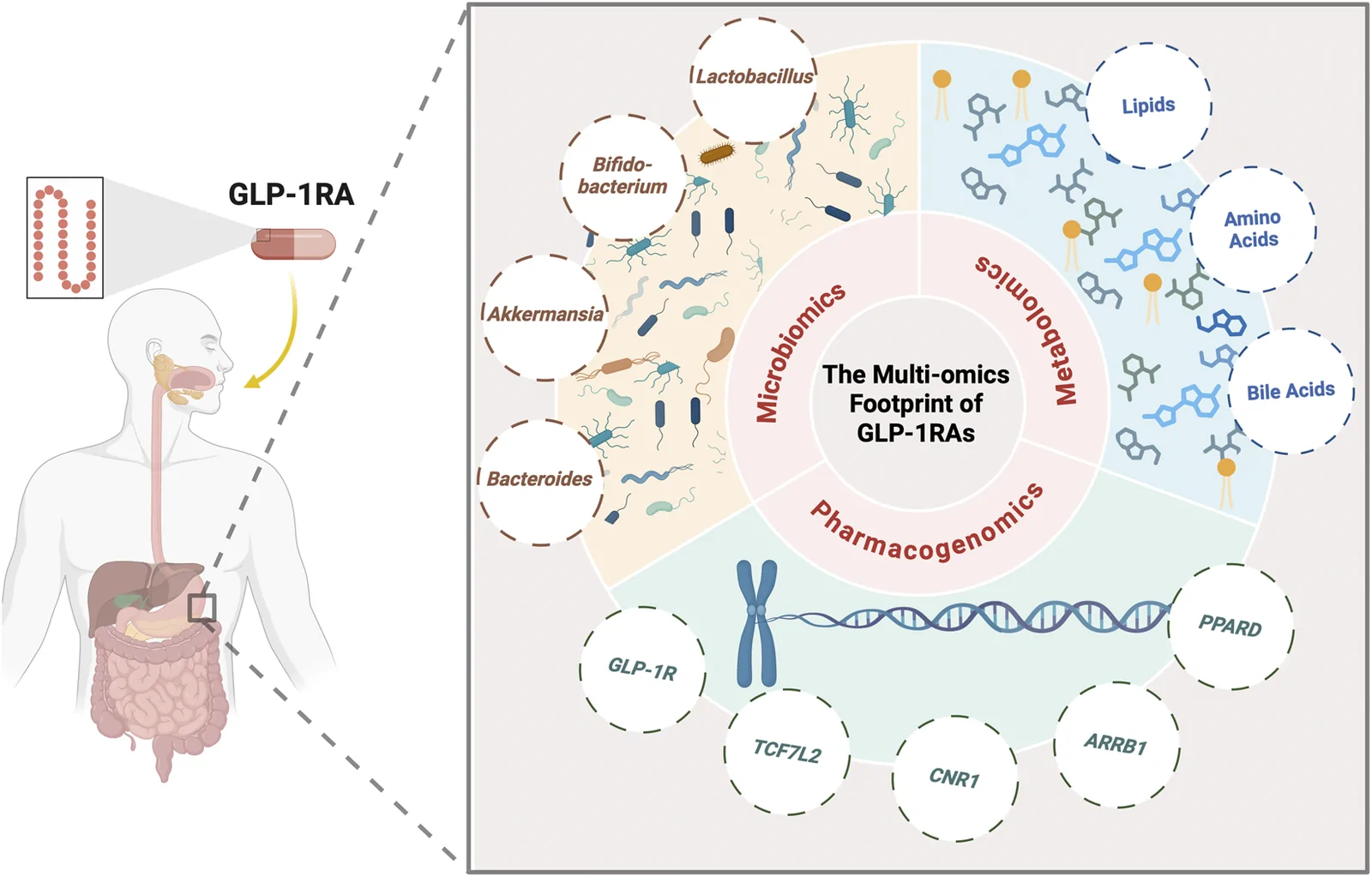
Created by BioRender.com.

## 1 Introduction

The global rise in type 2 diabetes (T2D) and obesity (NCD Risk Factor Collaboration, 2024; Phelps et al., 2024) underscores the critical need for effective and sustainable therapeutic strategies. While foundational, lifestyle interventions often prove insufficient for long-term glycemic and weight control (Mohammed et al., 2020; Caudwell et al., 2009; Khattab, 2024; Bailey, 2024), and bariatric surgery, though effective, carries inherent surgical risks (Wolfe et al., 2016). Conventional T2D treatments, such as metformin, sulfonylureas and insulin, often face limitations, including adverse effects, insufficient impact on diabetes-related complications, and inadequate potency to sustainably and safely control hyperglycemia in certain cases (Nabrdalik et al., 2022; Sayedali et al., 2023; Infante et al., 2021; Petznick, 2011; Brown et al., 2017; Costello et al., 2026; DeFronzo et al., 2013). These challenges underscore the therapeutic gap for agents capable of delivering robust glycemic and weight control while minimizing the risks of hypoglycemia and cardiovascular (CV) complications, driving the development and clinical adoption of glucagon-like peptide-1 receptor agonists (GLP-1RAs).

In 1992, John Eng et al. (Eng et al., 1992) isolated exendin-4, a 39-amino acid peptide, from the venom of the Gila Monster lizard *Heloderma suspectum*. This peptide aligned well with members of the glucagon superfamily, one of which is glucagon-like peptide-1 (GLP-1). GLP-1 is an incretin hormone that is secreted by the intestinal enteroendocrine cells and the brainstem preproglucagon neurons in response to food intake and other stimuli. It plays important roles in various physiological processes, such as glycemic control, satiety, electrolyte balance and cardiac function (Eng et al., 1992; Krieger et al., 2025). Like GLP-1, exendin-4 binds GLP-1 receptor (GLP-1R), mediating glucoregulatory activities; but with prolonged effect and higher potency, likely due to the longer half-life and higher bioavailability (Parkes DG. et al., 2001; Edwards et al., 2001; Goke et al., 1993; Kolterman et al., 2003; Young et al., 1999; Parkes D. et al., 2001). This inspired a series of clinical trials that tested the synthetic version of this peptide, Exenatide, for the treatment of T2D. Some trials used Exenatide as a monotherapy, while others added Exenatide to the treatment plan of T2D patients where diet and exercise or maximally effective doses of sulfonylureas and/or metformin failed to achieve optimal glycemic control. Across various studies, Exenatide improved glycemic indices, such as HbA1c and fasting plasma glucose levels, with minimal risk of severe hypoglycemia. It also reduced body weight compared to placebo, indicating additional benefits (Fineman et al., 2003; Buse et al., 2004; DeFronzo et al., 2005; Kendall et al., 2005; Poon et al., 2005).

In 2005, the U.S. Food and Drug Administration (FDA) approved the use of Exenatide for the treatment of T2D where metformin and sulfonylurea mono- or combined therapies fail to achieve adequate glycemic control (U.S. Food and Drug Administration Center for Drug Evaluation and Research, 2005a). This marked a key milestone that set up the stage for the development and FDA-approval of several other agents in the class, having yet a much higher homology to the human native GLP-1, and therefore collectively referred to as GLP-1 analogues. In 2014, Liraglutide was the first GLP-1RA to be approved by the FDA as an adjunct therapy for weight management, broadening the range of therapeutic applications of this class of drugs (U.S. Food and Drug Administration Center for Drug Evaluation and Research, 2014). Afterwards, the FDA has approved several GLP-1RAs as adjunct therapies to reduce the risk of major CV events, representing a significant advance in treating T2D, obesity, and associated comorbidities. Specifically, Liraglutide and Dulaglutide were initially approved for glycemic control in T2D, with demonstrated CV benefits (U.S. Food and Drug Administration Center for Drug Evaluation and Research, 2017; U.S. Food and Drug Administration Center for Drug Evaluation and Research, 2015; Gerstein et al., 2019). The latest approved GLP-1RA is semaglutide, which is available in injectable formulation for T2D and obesity or overweight with at least one associated complication, as well as in oral formulation for T2D (Australian Prescriber, 2025). In 2024, Semaglutide also became the first GLP-1RA approved for reducing serious CV events in patients with obesity and CV disease (U.S. Food and Drug Administration Center for Drug Evaluation and Research, 2024). Moreover, Tirzepatide is the first available agent from the new class of dual GLP-1/glucose-dependent insulinotropic polypeptide (GIP) receptor agonists, and it is approved for both T2D and obesity (U. S. Food and Drug Administration Center for Drug Evaluation and Research, 2022; U.S. Food and Drug Administration Center for Drug Evalua tion and Research, 2023).

The emergence and FDA-approval of GLP-1RAs have transformed how clinicians’ approach T2D treatment by addressing root causes of metabolic dysfunction as well as reducing complications. Beyond approved uses, new indications continue to expand. For instance, in 2025, Semaglutide received accelerated approval for treating metabolic dysfunction-associated steatohepatitis (MASH), making it the first GLP-1RA for this liver condition (U.S. Food and Drug Administration Center for Drug Evaluation and Research, 2025a). Moreover, Semaglutide received approval for reducing the risk of kidney disease progression (diabetic nephropathy) and cardiovascular death in chronic kidney disease and T2D patients (U.S. Food and Drug Administration Center for Drug Evaluation and Research, 2025b). Oral formulations of Semaglutide have also gained traction, with an FDA approval in late 2025 for chronic weight management and CV risk reduction, potentially becoming the first oral GLP-1RA for these indications (U.S. Food and Drug Administration Center for Drug Evaluation and Research, 2026).

Although GLP-1RAs have revolutionized the management of T2D and obesity through their metabolic and cardioprotective effects, individual responses to these agents vary considerably (Janez et al., 2022). Evidence now points to multiple factors, including genetic variation, clinical characteristics, metabolic signatures, and gut microbiome composition, as key determinants of both treatment response and side-effect profiles (Dawed et al., 2023; Babenko et al., 2019; Tsai et al., 2021). Recognizing and characterizing these interindividual differences is crucial for advancing a more personalized therapeutic approach. Integrating insights from genomics, metabolomics, and microbiome studies can help elucidate the biological mechanisms underlying response heterogeneity and guide more effective, individualized treatment strategies (Zhu et al., 2024). Such integration aligns with the broader goal of improving GLP-1RA utilization by maximizing efficacy, minimizing adverse effects, and informing future drug development. The present review will summarize current understanding of the mechanisms of action of GLP-1RAs and will focus on the genome-, metabolome-, and microbiome-derived determinants of GLP-1RAs response and tolerability. It will further discuss how integrating these complementary domains into a single precision medicine framework can refine clinical decision-making and contribute to the development of more effective GLP-1RA-based therapies, mainly in T2D and obesity.

## 2 Mechanism of action

### 2.1 GLP-1R signaling and molecular mechanisms

Upon binding of GLP-1 to GLP-1R, G-proteins are activated, leading to stimulation of adenylate cyclase and rising levels of intracellular cyclic AMP (cAMP). As a result, protein kinase A (PKA) and Ras-associated protein1 (Rap1) are activated, thereby contributing to the regulation of insulin and glucagon secretion. Moreover, phosphoinositide 3-kinase (PI3K)/protein kinase B (Akt) pathway is stimulated by the direct or indirect interaction of the activated GLP-1R with the regulatory subunits of PI3K. The activation of this pathway supports the survival and function of several cell types and tissues, including pancreatic β-cells, muscles, and adipose tissue. GLP-1 also activates AMP-activated protein kinase (AMPK) signaling in skeletal muscles, enhancing mitochondrial biogenesis and skeletal muscle remodeling. Furthermore, GLP-1 can act directly on hypothalamic receptors, activating neurons that promote satiety and decrease food intake (Zheng et al., 2024).

Aside from G-proteins, activated GLP-1R can recruit β-arrestin-1, which activates downstream signaling, such as cyclic amp response element-binding protein (CREB), extracellular signal-regulated kinase 1/2 (ERK1/2) and cAMP that are important for β-cell function and insulin secretion (Sonoda et al., 2008). β-arrestin-2 is also recruited to GLP-1R, but it has a dual role that may help maintain homeostasis. At physiological but not pharmacological concentrations of GLP-1, β-arrestin-2 reduces insulin secretion by partially uncoupling cAMP/PKA signaling. At pharmacological doses, however, β-arrestin-2 contributes to the GLP-1 mediated activation of CREB and ERK1/2 pathways (Zaïmia et al., 2023).

Like GLP-1, GLP-1RAs can activate GLP-1R and its downstream signaling pathways. In addition, some GLP-1RAs may show biased agonism. This is demonstrated by selective recruitment of some G-protein subtypes over others, as well as a preference for the G-protein coupled pathway with less recruitment of β-arrestin-2. GLP-1RAs characterized with the latter bias were associated with decreased GLP-1R internalization and recycling, explaining part of their mechanism of action in addition to evading the uncoupling effect of β-arrestin-2 (Wong et al., 2025).

GLP-1RAs have also been shown to affect genes and signaling pathways in ways that confer metabolic and non-metabolic benefits that extend beyond glucose regulation and body weight management. Among these are potential protective effects against fatty liver disease (Petrovic et al., 2023). For example, exendin-4 stimulates β-catenin signaling pathway which suppresses forkhead box protein A1 (FOXA1) and, subsequently, fatty acid-binding protein 1 (FABP1) expression levels. As a result, it decreases fatty acid uptake, mitigating steatosis in hepatocytes (Khalifa et al., 2022). Liraglutide, on the other hand, can enhance lipid clearance through transcription factor EB (TFEB)-mediated autophagy–lysosomal pathway, alleviating steatosis *in vitro* and *in vivo* (Fang et al., 2020). From a clinical perspective, GLP-1RAs were demonstrated to improve several indicators of liver disease, including liver fat content, inflammation, stiffness and liver enzymes (Wang et al., 2025).

Furthermore, GLP-1RAs have a well-established CV-protective role. This role can partially be attributed to indirect mechanisms that involve the improvement of lipid profiles, hyperglycemia and obesity. For example, Semaglutide and Dulaglutide may lower total cholesterol and low-density lipoprotein (LDL) cholesterol in T2D patients. In addition, Semaglutide and Exenatide can significantly reduce triglyceride levels (Janez et al., 2024). We have also previously reported that some GLP-1RAs with proven CV benefit, such as Liraglutide, have an anti-atherogenic effect by reducing the presence of pro-inflammatory and pro-atherogenic small, dense LDL (Nikolic et al., 2021). Indeed, small dense LDL are closely associated to the formation and the progression of atherosclerosis, representing a strong biomarker of CV risk (Mikhailidis et al., 2011).

In addition to the mentioned mechanisms, evidence indicate that GLP-1RAs may also act through direct mechanisms to support cardiac health (Park et al., 2024). For instance, under diabetic conditions, Liraglutide restores vascular endothelial function by improving angiogenesis, inhibiting apoptosis and decreasing oxidative stress (Rizzo et al., 2015). These effects are related to the enhancement of mitochondrial function in endothelial cells. Such enhancement could be explained by Liraglutide-mediated decrease in PTEN-induced kinase 1 (PINK1)/parkin expression with restoration of endothelial nitric oxide synthase (eNOS) phosphorylation and silent information regulator sirtuin 1 (SIRT1) expression (Zhang Y. et al., 2022). Additionally, Liraglutide can protect against reactive oxygen species (ROS) and adverse vascular effects in heart tissue of hypertensive models by suppressing NADPH oxidase (NOX) activity and recoupling of eNOS (Helmstädter et al., 2020). Another GLP-1RA, exendin-4, was shown to restore gene expression changes associated with glucolipotoxicity of endothelial cells, providing plausible mechanisms for its protective effects against atherosclerosis and CV events. For example, it enhances the expression of genes that play roles in vascular function and thrombosis, such as eNOS, plasminogen activator, tissue type (PLAT), platelet endothelial cell adhesion molecule (PECAM) and matrix protein fibronectin. Moreover, exendin-4 may prevent apoptosis as it balances the proapoptotic protein Bcl-2-associated X protein (Bax) with the antiapoptotic protein B-cell lymphoma-2 (Bcl-2) (Erdogdu et al., 2012). The canonical G-protein signaling, including cAMP/PKA and PI3K/Akt pathways, are suggested to be involved in the improvement of plaque stability and thrombosis as well as antioxidative and endothelial cell survival mechanisms of GLP-1RAs (Park et al., 2024).

Cardioprotection, as well as the enhancement of a broad spectrum of medical conditions, may also be mediated by the anti-inflammatory roles of GLP-1RAs. To illustrate, Exenatide polarizes macrophages toward the anti-inflammatory M2 phenotype, with a likely involvement of signal transducer and activator of transcription 3 (STAT3) activation (Shiraishi et al., 2012). It can also reverse systemic inflammation by restoring the balance between T helper 17 (Th17) and regulatory T (Treg) cell populations, an effect which is at least partially mediated by the PI3K/Akt/forkhead box O1 (FoxO1) pathway (Xu et al., 2022). Besides, GLP-1RAs induce organ-specific changes in immune cell activation, proliferation and/or migration, providing a therapeutic potential for different medical conditions by reducing inflammation (Helmstädter et al., 2020; Xu et al., 2022; Moschovaki Filippidou et al., 2020; Gharagozloo et al., 2021). Consistent with that, GLP-1RAs significantly decrease serum levels of the pro-inflammatory markers C-reactive protein (CRP), monocyte chemoattractant protein-1 (MCP-1), tumor necrosis factor-alpha (TNF- α), interleukin-6 (IL-6), interleukin-1 beta (IL-1β) and leptin while increasing the anti-inflammatory marker adiponectin (Ren et al., 2025; Ha et al., 2024; Liu J. et al., 2022). This anti-inflammatory effect is largely driven through AMPK-mediated suppression of nuclear factor kappa-light-chain-enhancer of activated B cells (NF-κb) pathway, which is thought to contribute to multiple inflammation-associated conditions, such as endothelial dysfunction, ovarian inflammation, recognition memory dysfunction, and neurodegenerative diseases (Park et al., 2024; Liu et al., 2024; Cao et al., 2022; Zhang LQ. et al., 2021; Zhai et al., 2025).

### 2.2 Structural advantages of GLP-1RAs

Early clinical studies have shown that the administration of the native GLP-1 peptide enhances insulin release, inhibits glucagon secretion, decreases blood glucose and delays gastric emptying in individuals both with and without diabetes mellitus (Liu et al., 2024; Nauck et al., 1996; Ritzel et al., 1995; Willms et al., 1996; Schirra et al., 1997; Todd et al., 1997). In addition, it reduces appetite, energy intake and postprandial ghrelin levels (Näslund et al., 1999). However, this peptide has a short half-life due to the rapid cleavage by dipeptidyl peptidase-4 (DPP-4), which removes a dipeptide out of the N-terminal end of GLP-1, rendering it inactivated (Parkes D. et al., 2001; Ahrén, 2019). GLP-1RAs were designed to overcome this challenge by leveraging structural features that provide greater stability than the native peptide.

Some GLP-1RAs have structures that are very similar to the native GLP-1 peptide (GLP-1 analogues), with modifications that hinder DPP-4-mediated degradation but maintain GLP-1’s metabolic activity. This includes Liraglutide, Semaglutide, Albiglutide and Dulaglutide (Larsen et al., 2001; Lau et al., 2015; Glaesner et al., 2010; Bush et al., 2009). Liraglutide and Semaglutide are additionally attached to fatty acid moieties via different linkers, strengthening their binding to human albumin, which delays their renal excretion and increases their half-lives (Larsen et al., 2001; Lau et al., 2015). Albiglutide (discontinued due to economic reasons) is directly fused to human albumin. Thus, it was designed with a GLP-1 analogue dimer instead of a monomer to increase its ability to bind GLP-1R in the presence of albumin that could interfere with binding (Bush et al., 2009). Dulaglutide utilizes a different approach which involves linking GLP-1 analogue to a human immunoglobulin G4 constant region variant (IgG4-Fc). This modification reduces renal clearance by increasing the compound size. Additionally, it takes advantage of the FcRn salvage pathway, evading lysosomal degradation and extending the presence of IgG4-Fc fusion protein in the circulation (Glaesner et al., 2010; Geiser et al., 2016; Baldwin et al., 2019). Together, the structural designs of these drugs make them intermediate to long acting and allow for longer separation time between dosages that can extend to once weekly (Geiser et al., 2016).

In contrast to the previously described agonists, Exenatide is a GLP-1RA with much lower structural similarity to the native GLP-1 peptide as it has only 53% sequence identity (Eng et al., 1992; Geiser et al., 2016). This distinction confers resistance to DPP-4-mediated degradation due to an altered recognition site which prevents detection and cleavage by DPP-4 (Drucker, 2001). As a result, Exenatide’s half-life exceeds that of the native GLP-1. However, it is still shorter than the long-acting forms of GLP-1RAs, primarily due to the rapid elimination by renal clearance (Gao and Jusko, 2012). Accordingly, the first approved version of Exenatide was given in a twice daily format (U.S. Food and Drug Administration Center for Drug Evaluation and Research, 2005b). However, an injectable extended-release formulation has been later developed and approved by the FDA (discontinued due to economic reasons) (U.S. Food and Drug Administration Center for Drug Evaluation and Research, 2012). This formulation depends on biodegradable poly (lactide-co-glycolide) coating which slowly disintegrates, allowing for the gradual release of the encapsulated Exenatide and a once-weekly administration (Cai et al., 2013). Lixisenatide, another FDA-approved GLP-1RA, was developed by structural modifications of Exenatide, which gives it more stability and high affinity to GLP-1R, enabling a once-daily dosing (Barnett, 2011; Werner et al., 2010; U.S. Food and Drug Administration Center for Drug Evaluation and Research, 2016), although Liraglutide has shown a greater efficacy than Lixisenatide in improving glycemic control (Nauck et al., 2016).

We wish to highlight that GLP-1RAs with higher homology to the native human GLP-1, such as Liraglutide and Semaglutide compared with Exenatide and Lixisenatide, are generally more effective, especially for glycemic control (Trujillo et al., 2021). However, exceptions exist, such as the superior efficacy of Lixisenatide over Liraglutide in controlling postprandial glucose levels (Meier et al., 2015). While more homologous GLP-1RAs were also associated with less production of antibodies, the safety and efficacy of the less homologous agonists did not seem to be significantly affected by the presence of antibodies (Madsbad, 2016). Furthermore, the relationship between sequence homology, receptor affinity and clinical efficacy is not straightforward and some other factors play a role in this dynamic. For example, changes of key residues in the peptide backbone and attachment of carriers can modulate receptor affinity, ligand kinetics, downstream signaling, and drug absorbance, stability and clearance, ultimately effecting clinical efficacy (Knudsen and Lau, 2019; Werner, 2014; Zhang X. et al., 2021).

In addition to mono-agonists, research is advancing on dual and triple agonists targeting multiple receptors. Among these, only Tirzepatide is FDA approved (U. S. Food and Drug Administration Center for Drug Evaluation and Research, 2022; U.S. Food and Drug Administration Center for Drug Evalua tion and Research, 2023), but several other agents in are under development (Muzurovic et al., 2026). Tirzepatide is a dual agonist with a 39-amino acid peptide backbone, designed to activate GIP receptor (GIPR) and GLP-1R, while resisting DPP-4 degradation. The peptide is conjugated to a fatty diacid moiety which enhances binding to albumin, increasing its half-life and allowing for once-weekly dosing (Coskun et al., 2018). GIP is an insulinotropic incretin, overlapping with GLP-1 in certain biological activities, but differing in others. In line with that, GLP-1R/GIPR dual agonism by Tirzepatide induces more potent effects than mono-agonists, such as Semaglutide and Dulaglutide, especially on body weight. Moreover, it could maintain activities even with genetic deletion of either receptor (Coskun et al., 2018; Okuma, 2025). No other multi-agonist has been approved by the FDA yet, but some are in clinical trials, such as, but not limited to, CT-868, VK2735 (dual GLP-1/GIP receptor agonists), Survodutide, Mazdutide, Pemvidutide (dual GLP-1/glucagon receptor agonists) and Retatrutide (triple GLP-1/GIP/glucagon receptor agonist) (Chakravarthy et al., 2025; Çetin et al., 2025).

Overall, the structural differences and receptor engagement profiles of the indicated GLP-1RAs affect the agonist stability, its binding affinity to GLP-1R, the downstream signaling, and GLP-1R trafficking (Pickford et al., 2020). As a result, each GLP-1RA may elicit distinct biological responses that can shape the multi-pharmaco-omics profiles and affect the outcome of the treatment. The heterogeneity in these profiles, including the genomic, metabolomic, and microbiomic signatures are discussed in subsequent sections.

## 3 Safety and clinical tolerability of GLP-1RAs

While effective in improving glycemic control and supporting weight reduction, the safety of GLP-1RAs profile remains heterogeneous across studies. Gastrointestinal (GI) disturbances such as nausea, abdominal pain, constipation, diarrhea, and vomiting are the most frequently reported adverse effects (Liu L. et al., 2022). However, several studies have observed minimal or no clinically significant GI complications (Zhou et al., 2022), possibly reflecting differences in study design, population demographics, and treatment protocols.

Population-based data from the “All of Us” Research Program cohort (Aldhaleei et al., 2024) emphasized that demographic factors can modulate adverse event risk. Among 10,328 newly initiated users of varying ethnic backgrounds, abdominal pain was the most common adverse effect (57.6%, 95% CI: 56.6%–58.5%), while GI bleeding occurred more frequently in Dulaglutide and Liraglutide users. Gastroparesis was reported primarily in patients with chronic kidney disease using Liraglutide, Dulaglutide, or Exenatide. Differences by ethnicity and sex were notable; white users exhibited higher rates of diarrhea, nausea, and vomiting, while constipation was more prevalent among black users. Older men reported fewer GI effects than women and younger counterparts.

Beyond GI manifestations, emerging evidence highlights an increased risk of gallbladder and biliary diseases with GLP-1RA therapy. A meta-analysis of 76 randomized trials (He et al., 2022) reported relative risks of 1.37 overall, rising to 1.56 with higher doses, 1.40 with longer treatment durations, and 2.29 in weight-loss trials. Consistently, a large real-world study (Hurwitz et al., 2025) confirms an elevated risk of hospitalization for gallbladder and biliary conditions among GLP-1RA users.

In rodents, Liraglutide induced dose- and treatment-duration-dependent thyroid C-cell tumors (Madsen et al., 2012). Nonetheless, the relevance of this preclinical finding to humans remains uncertain. A recent observational study did not identify an increased risk of thyroid cancer among new users of GLP-1RAs compared with users of other glucose-lowering medications (Morales et al., 2025). GLP-1RAs-associated weight loss may also be accompanied by reductions in lean mass; however, current evidence is insufficient to establish that these agents directly cause sarcopenia (Pantazopoulos et al., 2025). Additionally, acute pancreatitis has been reported in patients receiving GLP-1RAs. A recent study found no increased risk of pancreatitis over up to 5 years among patients with T2D treated with GLP-1RAs (Ayoub et al., 2025).

Taken together, these findings indicate that while GLP-1RAs remain valuable therapeutic agents, clinicians should consider individual factors including age, sex, ethnicity, baseline nutritional and muscle status, comorbidities, prior medications, treatment duration, and dose, when evaluating GLP-1RAs tolerability and safety. Clinical monitoring is warranted for older adults and other individuals at risk of frailty or sarcopenia, as well as for those with gastrointestinal symptoms or risk factors for biliary complications.

## 4 Pharmacogenomics of GLP-1RAs

Pharmacogenomics hold considerable potential to improve treatment individualization through genetic profiling. Available evidence suggests that genetic polymorphisms among individuals may influence the pharmacokinetics and pharmacodynamics of GLP-1RAs drugs, thereby contributing to interindividual variability in treatment efficacy and susceptibility to adverse effects (Jakhar et al., 2022; Lin et al., 2015). The following section provides a comprehensive overview of how various genetic polymorphisms within genes associated with GLP-1RAs may affect drug response to different clinical traits. Table 1 and Figure 1, summarize the list of variants associated with GLP-1RA interventions. Variation across studies should be considered when interpreting these results. Differences in ancestry and linkage disequilibrium patterns, baseline phenotype and disease severity, use of other glucose-lowering medications, GLP-1RA agent, dose and duration of exposure, and definitions of treatment response may all contribute to the inconsistent findings.

**TABLE 1:** Pharmacogenomics associated with GLP-1RAs Intervention.

Gene	Variant(s)	Population/sample	GLP-1RA/intervention	Direction of effect/key findings	Ref
***GLP1R***	rs10305492 (A316T), rs146868158 (R421W)	*In vitro* functional studies	Multiple GLP-1RAs	A316T: increased GLP-1R activation and endocytosis to most ligands, including Tirzepatide R421W: opposite effect; ∼10-fold difference in receptor activation between A316T vs. R421W with Tirzepatide	(Lagou et al., 2023)
	rs5875654 (in LD with rs761386 C>T) rs3765467	Poorly controlled T2D patients	Exenatide + insulin	rs761386 CT/TT: higher 120-min OGTT glucose vs. CC despite normal C-peptide/insulin; significance lost after multiple testing	(Lin et al., 2015)
				rs3765467: no significant plasma glucose change	
	rs3765467	T2D patients	Exenatide or Liraglutide + metformin with or without Dapagliflozin	rs3765467 GG: greater HbA1c reduction vs. GA/AA (1.7% ± 2.4% vs. 0.8% ± 1.8%), no fasting glucose difference; stronger HbA1c effect in men with BMI ≥28 kg/m^2^ and HbA1c < 9%	(Guan et al., 2022)
	rs10305420 rs6923761	Obese women with polycystic ovary syndrome (PCOS)	Liraglutide	Strong responders lost more visceral fat than poor responders; rs10305420 CT/TT: reduced treatment efficacy vs. CC (OR 0.27, 95% CI 0.09–0.85) rs6923761 GA/AA: trend to stronger response vs. GG (OR 3.06, 95% CI 0.96–9.74, *p* = 0.058).	(Jensterle et al., 2015)
	rs6923761	Overweight T2D patients	Liraglutide	rs6923761 GA/AA: greater weight loss and better glycemic response vs. GG in some studies. However, several other studies report no significant association with weight loss or HbA1c	(Dawed et al., 2023; Eghbali et al., 2024; de Luis et al., 2015; Maselli et al., 2022)
	rs10305420	Overweight T2D patients	Exenatide	T allele carriers: less HbA1c reduction (*p* = 0.002) and less weight loss (*p* = 0.02); strong correlation between HbA1c reduction and weight loss (each 1% HbA1c drop ≈0.52 kg weight loss)	(Yu et al., 2019)
	rs10305420	T2D patients	Exenatide or Liraglutide	No significant HbA1c differences between CT/TT vs. CC genotypes	(Guan et al., 2022)
	rs10305420	T2D patients	Liraglutide	TT genotype: significantly better glycemic response vs. CC/CT (recessive model: OR 3.28, 95% CI 1.41–7.65; codominant model: OR 2.52, 95% CI 1.03–6.13)	(Eghbali et al., 2024)
***TCF7L2***	rs7903146	Healthy obese individuals	Liraglutide	CC genotype: lower body weight at 16 weeks vs. CT/TT, but absolute weight changes similar between genotypes	(Maselli et al., 2022)
	rs7903146	Overweight or obese T2D	Exenatide	CT/TT: higher pre-treatment insulin and proinsulin. However, after Exenatide treatment CT/TT showed greater reduction in postprandial insulin/proinsulin	(Ferreira et al., 2019)
	rs7903146	Meta-analysis	Various GLP-1RAs	T allele associated with better glucose control in some cohorts but inconsistent Pooled meta-analysis showed moderate but non-significant effect on weight loss	(Kyriakidou et al., 2024; BouSaba et al., 2023)
	rs7903146	Obese patients	Exenatide	CT/TT carriers: significantly greater weight loss vs. CC (1.65 ± 1.03 kg vs. −0.47 ± 0.66 kg)	(BouSaba et al., 2023; Chedid et al., 2018)
***CNR1***	rs1049353	T2D patients	Liraglutide	GG: larger reductions in total and LDL cholesterol AA: greater HOMA-IR reduction; A allele associated with more favorable metabolic profile overall	(de Luis et al., 2014)
***ARRB1***	rs140226575	Multi-ethnic GLP-1RA users	GLP-1RAs	Carriers of *ARRB1* rare variants, driven by rs140226575, showed significantly greater HbA1c reduction vs. non-carriers. The effect was consistent across ethnic groups	(Dawed et al., 2023)
***PPARD***	rs3777744	Newly diagnosed T2D patients (n = 105)	Exenatide monotherapy, 6 months	AA genotype associated with a higher responder rate and greater HbA1c improvement vs. AG/GG.	(Song et al., 2022)
	rs2016520			c-allele carriers had lower post-treatment HOMA-IR than TT carriers	(Song et al., 2022)
***CTRB1/2***	rs7202877	Non-diabetic individuals (n = 232)	Intravenous GLP-1 during hyperglycaemic clamp	G allele: ∼33% higher insulin secretion upon GLP-1 stimulation vs. TT.	(t Hart et al., 2013)
	rs7202877	T2D patients (n = 173)	Intravenous GLP-1 during hyperglycaemic clamp	No significant association with HbA1c response to GLP-1RA therapy in T2D (*p* = 0.58)	(t Hart et al., 2013)
	rs7202877	T2D patients	Liraglutide, Exenatide, Lixisenatide	No significant association with HbA1c or weight-loss response to GLP-1RAs across the available cohorts	(Kyriakidou et al., 2022)
***SORCS1***	rs1416406	Newly diagnosed T2D (n = 101)	Exenatide	GG genotype associated with a significantly greater improvement in the proinsulin-to-insulin ratio than GA/AA genotypes; no reported genotype-specific differences in broader glycaemic outcomes	(Zhou et al., 2017)
***WFS1***	c.316-1G>A and other WFS1 variants	Wolfram syndrome models: patient iPSC-derived β cells and WFS1-deficient mice	Liraglutide, Exenatide, Dulaglutide	Liraglutide restored Ca^2+^ oscillations and enhanced glucose-stimulated insulin secretion in *WFS1*-mutant iPSC-derived β cells; Exenatide and Dulaglutide improved β-cell function and survival in *WFS1*-deficient human models; Dulaglutide reversed impaired glucose tolerance in Wfs1-deficient mice	(Torchio et al., 2025; Gorgogietas et al., 2023)

**FIGURE 1:**
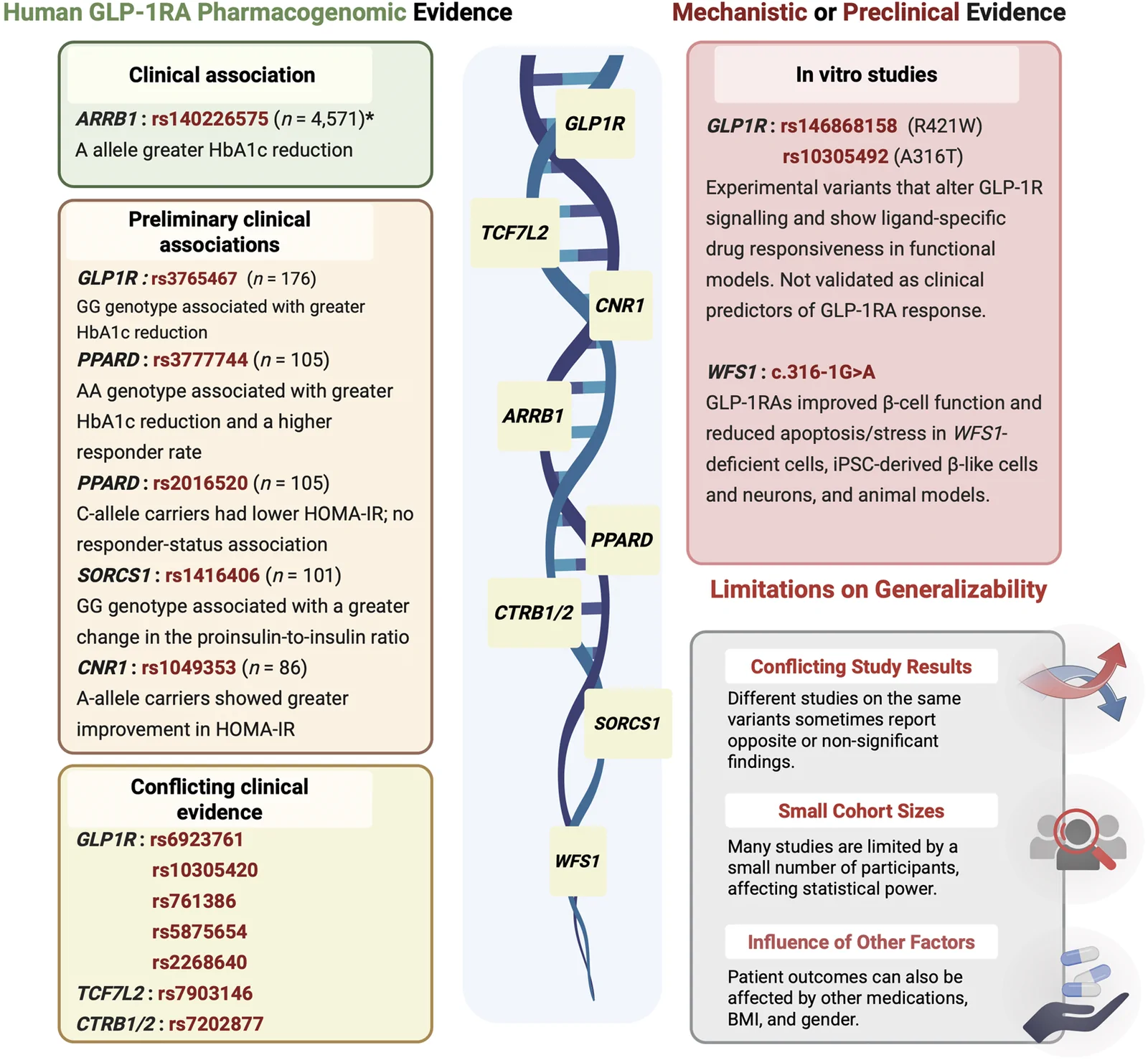
Summary of pharmacogenomic evidence for genetic determinants of GLP-1RA treatment response. Human evidence is categorized as a clinical association, preliminary clinical associations, or conflicting clinical evidence according to the consistency and clinical relevance of available findings. All findings classified as preliminary clinical associations derive from single cohorts and have not undergone independent replication. Conflicting clinical evidence includes variants with null, inconsistent, or insufficiently confirmed associations with GLP-1RA glycaemic or weight responses. (*) denotes an association with independent replication. Sample sizes of treated study populations are shown in parentheses (n). The mechanistic/preclinical panel does not represent validated clinical pharmacogenomic predictors of GLP-1RA response. Detailed study characteristics, outcomes, and replication status are provided in Table 1. Created by BioRender.com.

### 4.1 GLP1R

Genetic variability within the GLP-1 receptor (*GLP1R*) gene has been associated with dysregulation in receptor function (Beinborn et al., 2005), pancreatic insulin secretion, and response to receptor agonists (Koole et al., 2011). Several studies elucidated the disruptive effects of *GLP1R* variants on pancreatic β-cell glucose-stimulated insulin secretion, β-cell responsiveness and apoptosis (Li et al., 2020; Sathananthan et al., 2010). Building on these findings, *in vitro* studies examined the effects of endogenous GLP-1 and pharmacological GLP-1RAs on activation and endocytosis of selected GLP-1R coding variants previously associated with random glucose levels (Dawed et al., 2023). For instance, rs10305492 (A316T) and rs146868158 (R421W) variants demonstrated distinct responses to most tested agonists. A316T exhibited increased GLP-1R activation and endocytosis in response to nearly all tested ligands *in vitro*, including the dual agonist Tirzepatide, whereas R421W displayed opposite response. Notably, upon treatment with Tirzepatide, the GLP-1R activation differed by nearly tenfold between A316T and R421W (Lagou et al., 2023). In contrast, a recent study investigated the clinical characteristics of A316T. Interestingly, the mutation was found to have improved glucose homeostasis but had lower drug responsiveness to GLP-1RAs *in-vitro* (El Eid et al., 2026).

#### 4.1.1 Rs761386 and rs5875654

To further investigate the genetic associations of GLP-1RAs, a study by Lin *et al.* genotyped the coding regions of *GLP1R* in 36 poorly controlled T2D patients who received subcutaneous insulin infusions for 3 days, followed by dual treatment with subcutaneous insulin infusions and Exenatide for additional 3 days (Lin et al., 2015). This resulted in the identification of variants including rs5875654 which is in a strong linkage disequilibrium (LD) with rs761386 C>T. Both variants were significantly associated with changes in the standard deviation of plasma glucose levels in T2D patients (Lin et al., 2015). However, these associations did not remain significant after correction for multiple testing (Lin et al., 2015), and should therefore be replicated in a larger cohort.

#### 4.1.2 Rs3765467

The rs3765467 variant was nominally associated with changes in the standard deviation of plasma glucose levels in the Lin *et al.* study, however, this association did not remain significant after adjustment for multiple testing (Lin et al., 2015), In a prospective Chinese cohort of 176 participants with T2D treated with Exenatide or Liraglutide for 12 weeks, HbA1c reduction was greater in participants with the rs3765467 GG genotype than in GA + AA carriers (1.7% ± 2.4% vs. 0.8% ± 1.8%, *p* = 0.002) (Guan et al., 2022). The proportion of patients who achieved the HbA1c target of <7.0% was also higher among GG carriers (50.9% vs. 23.8%, *p* = 0.002). However, no significant genotype-related differences were observed in fasting plasma glucose or BMI reduction. Furthermore, in subgroup analyses, the HbA1c association was statistically significant among men, participants with a baseline BMI ≥28 kg/m^2^, and those with baseline HbA1c <9%, but not in the corresponding comparison groups (Guan et al., 2022). In this study, participants were also receiving other medications including metformin with or without dapagliflozin (Guan et al., 2022), which may have influenced treatment response. Overall, rs3765467 may be associated with glycaemic response to GLP-1RA treatment, particularly HbA1c reduction, however, the evidence remains preliminary and requires independent replication.

#### 4.1.3 Rs10305420

Several studies have elucidated the genetic variability of *GLP1R*, specifically rs10305420 in response to GLP-RAs treatments. In 57 obese women with polycystic ovary syndrome (PCOS) treated with Liraglutide for 12 weeks, carriers of the CT or TT genotypes showed significantly lower treatment efficacy than CC carriers (OR = 0.27, 95% CI = 0.09–0.85), despite significant reductions in weight, BMI, fasting glucose, and OGTT glucose in both responder groups (Jensterle et al., 2015). Similarly, in 285 overweight patients with T2D treated with Exenatide for 6 months, the T allele was associated with less HbA1c reduction (*p* = 0.002) and lower weight loss (*p* = 0.02), and each 1% decrease in HbA1c corresponded to an average weight loss of 0.52 kg (Yu et al., 2019). On the contrary, a recent study on Iranian patients with T2D carrying the rs10305420 TT genotype demonstrated a significantly better glycemic response compared to those with CC or CT genotypes (recessive model: OR 3.28, 95% CI 1.41-7.65; codominant model: OR 2.52, 95% CI 1.03–6.13) following 6 months of Liraglutide treatment (Eghbali et al., 2024). However, no significant HbA1c difference was observed in Chinese cohorts treated with Exenatide or Liraglutide (Guan et al., 2022; Yu et al., 2019) and a similar result were reported in a meta-analysis study (Dawed et al., 2023).

#### 4.1.4 Rs6923761

The rs6923761 variant has also been linked to GLP-1RA response. Overweight patients with T2D carrying the rs6923761 GA or AA genotypes exhibited greater weight reduction (*p* < 0.05) after 14 weeks of Liraglutide treatment compared to patients with the GG genotype (de Luis et al., 2015). In addition, a significant association was also reported between rs6923761 and glycemic response (Dawed et al., 2023). However, the association was not consistently replicated. In the PCOS Liraglutide study, GA/AA carriers showed a nonsignificant trend toward greater response than GG carriers (Jensterle et al., 2015) and other studies found no significant association with weight loss or HbA1c change (Eghbali et al., 2024; Maselli et al., 2022).

#### 4.1.5 Additional variants in *GLP1R*

A genome-wide association study identified a polymorphism, rs2268640 (*p* = 2.5 × 10^−7^), associated with glycemic response in GLP-1RAs users (Dawed et al., 2023). However, evidence for its clinical relevance remains limited. Another variant of interest, rs367543060 could not be assessed due to the absence of participants with heterozygous or homozygous alternative alleles (Kyriakidou et al., 2024).

Taken together, these findings suggest that genetic variation in *GLP1R* may contribute to interindividual differences in response to GLP-1RAs, particularly with respect to glycemic and weight outcomes. Among the reported variants, rs10305420, rs6923761, rs3765467, and the rs761386/rs5875654 locus have shown potential associations with treatment response, but the results remain inconsistent across studies and populations, and most evidence is still preliminary. Therefore, these variants are promising candidates for pharmacogenetic stratification, but further validation in larger, well-characterized cohorts is needed before clinical application.

### 4.2 TCF7L2

Studies have investigated the effects of GLP-1RAs and the pharmacogenomic interactions of the *TCF7L2* genetic variant, rs7903146, on weight, gastric function and postprandial plasma insulin, and proinsulin levels. Evidence for *TCF7L2* rs7903146 is suggestive but not definitive. In 59 adults with obesity, Liraglutide for 16 weeks delayed gastric emptying of solids and increased weight loss compared to placebo. Moreover, CC carriers were associated with lower body weight than the CT/TT carriers post treatment (*p* = 0.015) (Maselli et al., 2022). However, no significant association between the rs7903146 and change in weight were observed (Maselli et al., 2022; BouSaba et al., 2023). Similar outcomes were also observed in several studies including overweight and obese patients with T2D treated with Exenatide for 8 weeks (Ferreira et al., 2019) or in 10 obese patients Exenatide for 4 weeks (BouSaba et al., 2023; Chedid et al., 2018). or in T2D patients on GLP-1RAs (Kyriakidou et al., 2024) and in a meta-analysis where only a moderate, non-significant association with weight loss and rs7903146 (BouSaba et al., 2023). Nonetheless Exenatide treatment on patients with CT or TT genotypes showed a significant reduction in postprandial plasma insulin, and proinsulin levels (*p* < 0.05) compared to CC genotype carriers (Ferreira et al., 2019). These results indicate that *TCF7L2* may influence aspects of metabolic response to GLP-1RAs, but current evidence does not support a strong or consistent predictive effect on weight loss.

### 4.3 CNR1

De Luis *et al.* investigated rs1049353, a common variant in the cannabinoid type 1 receptor gene (*CNR1*), in 86 patients with T2D and poor glycemic control who were receiving metformin or sulfonylurea and were subsequently treated with Liraglutide for 14 weeks. Participants with the GG genotype had higher BMI, body weight, fat mass, and waist circumference than AA or GA carriers at baseline and after treatment. Liraglutide improved glycemic control and HbA1c levels irrespective of genotype. However, significant reductions in total cholesterol and LDL cholesterol were observed only in patients with the GG genotype, whereas improvement in IR, assessed by HOMA-IR, was observed only in A-allele carriers (de Luis et al., 2014). Thus, rs1049353 was associated with differential, outcome-specific metabolic changes following Liraglutide treatment, rather than a consistent overall metabolic advantage of the A allele. Given the small single-cohort design, these findings require independent replication.

### 4.4 ARRB1

A genome-wide gene-based analysis identified an association between low-frequency and rare coding variants in the *ARRB1* gene and glycaemic response to GLP-1RA therapy (SKAT-O *p* = 6.7 × 10^−8^) (Dawed et al., 2023). This association was largely driven by the low-frequency rs140226575 G>A (Thr370Met) variant. Each rs140226575 A allele was associated with a significantly greater HbA1c reduction of 0.25% (2.7 mmol/mol; *p* = 5.2 × 10^−6^) (Dawed et al., 2023). The rs140226575 A allele was more prevalent in Hispanic and American Indian or Alaska Native populations than in White European populations, however, its association with glycemic response was directionally consistent across ancestry-stratified analyses (Dawed et al., 2023). The rs140226575 association was subsequently replicated in an independent GLP-1RA-treated dataset from the HARMONY outcomes trial (*p* = 2.3 × 10^−4^) within the same study (Dawed et al., 2023). However, further validation in diverse populations and studies assessing clinical outcomes after GLP-1RA treatment are warranted.

### 4.5 PPARD

A study by Song *et al.* investigated the effects of two *PPARD* gene variants, rs3777744 and rs2016520, on response to 6 months of Exenatide monotherapy in 105 newly diagnosed T2D patients.

#### 4.5.1 Rs3777744

Following Exenatide treatment, patients exhibited significant improvements in multiple clinical parameters, including BMI, fasting plasma glucose, HbA1c, triglycerides, low-density LDL-C, and HOMA-IR. Patients were classified as responders if they achieved either an HbA1c reduction of at least 1.0% or body-weight loss of at least 3% loss. Notably, carriers of the rs3777744 AA genotype had a significantly higher response rate than carriers of the AG or GG genotypes (84.0% vs. 61.36% and 54.55%, respectively; *p* = 0.022) (Song et al., 2022). In multivariable analysis, rs3777744 was significantly associated with HbA1c improvement after Exenatide treatment (*p* = 0.009) (Song et al., 2022).

#### 4.5.2 Rs2016520

For rs2016520, carriers of at least one C allele had significantly lower HOMA-IR levels after 6 months of Exenatide treatment than participants with the TT genotype (*p* < 0.05) (Song et al., 2022). However, rs2016520 was not significantly associated with responder status based on the predefined Exenatide-response criteria (genotype *p* = 0.584; allele *p* = 0.182).

These findings suggest that rs3777744 and rs2016520 may influence distinct components of Exenatide response; however, replication in independent and ancestrally diverse cohorts is needed, as the current evidence is based on a single Chinese cohort of Exenatide-treated patients with T2D.

### 4.6 CTRB1/2

The *CTRB1/2* locus, specifically the rs7202877 variant, was significantly associated with GLP-1-stimulated insulin secretion (*p* ≤ 8.8 × 10^−7)^ during hyperglycemic clamps in 232 healthy Caucasian non-diabetic individuals. Carriers of the G allele exhibited a 33% increase in insulin secretion upon GLP-1 stimulation (*p* = 1.9 × 10^−6^) (t Hart et al., 2013). However, this physiological association did not translate into a significant difference in HbA1c response to GLP-1RA therapy among patients with T2D. In contrast, among DPP-4 inhibitor-treated patients, G-allele carriers had a smaller reduction in HbA1c than TT carriers in a meta-analysis of two cohorts (*p* = 0.0015) (t Hart et al., 2013). Thus, rs7202877 may be associated with endogenous incretin physiology and DPP-4 inhibitor response, but available evidence does not support an association with clinical response to GLP-1RAs.

Kyriakidou *et al.* further evaluated rs7202877 in patients with T2D treated with Liraglutide for at least 6 months and found no significant association with either glycemic response (*p* = 0.69) or weight-loss response (*p* = 0.84) (Kyriakidou et al., 2022). Additionally, among weight-loss non-responders, Liraglutide treatment was associated with a significant reduction in HbA1c, whereas changes in weight, BMI, and fasting plasma glucose were not statistically significant (Kyriakidou et al., 2022). Genotypic analysis was limited by the absence of participants with the GG genotype (Kyriakidou et al., 2022). Consistent with these findings, a subsequent study of 191 Greek patients with T2D treated with Liraglutide, Exenatide, or Lixisenatide found no significant association between rs7202877 and glycaemic response (*p* = 0.47) or weight-loss response (*p* = 0.57) (Kyriakidou et al., 2024). Overall, these two studies were conducted in Greek populations (Kyriakidou et al., 2024; Kyriakidou et al., 2022), and the available clinical evidence does not support rs7202877 as a predictor of glycaemic or weight-loss response to GLP-1RA therapy.

### 4.7 SORCS1

The *SORCS1* rs1416406 variant has been evaluated in relation to β-cell functional responses to Exenatide treatment (Zhou et al., 2017). Zhou *et al.* investigated the relationship between the rs1416406 variant with glucose response and pancreatic β-cell function after 48 weeks of Exenatide treatment in newly diagnosed T2D patients (n = 101). Across the cohort, Exenatide treatment was associated with significant improvements in clinical traits including BMI, HbA1c, fasting blood glucose, and β-cell function measures, such as proinsulin/insulin ratio, disposition index, and acute insulin response. Notably, genotype-stratified analysis showed that patients carrying the rs1416406 GG genotype exhibited a significantly greater improvement only in the proinsulin-to-insulin ratio than carriers of the GA and AA genotypes. This association remained significant (*p* < 0.05) after adjustment for age, sex, baseline BMI, HbA1c, and proinsulin-to-insulin ratio (Zhou et al., 2017). Thus, rs1416406 represents a preliminary, outcome-specific association with an Exenatide-related β-cell function marker rather than a replicated predictor of overall glycaemic or weight-loss response to GLP-1RA therapy.

### 4.8 WFS1

A recent study by Torchio *et al.* reported that *WFS1* variants, including c.316-1G>A, are associated with early-onset diabetes and a chronic inflammatory state in Wolfram syndrome. In patient-derived iPSC β-cells harboring the c.316-1G>A variant, acute Liraglutide treatment restored Ca^2+^ oscillations and enhanced glucose-stimulated insulin secretion compared with untreated cells (Torchio et al., 2025). In complementary preclinical models, Dulaglutide reversed impaired glucose tolerance in *WFS1*-deficient mice. Both Exenatide and Dulaglutide improved pancreatic β-cell function and reduced apoptosis in *WFS1*-deficient human β-cells and patient-derived iPSCs models (Gorgogietas et al., 2023). Collectively, these findings support the potential of GLP-1RAs to ameliorate mutation-associated β-cell dysfunction in Wolfram syndrome. Importantly, these are disease-model rescue findings from patient-derived cells and animal models, rather than population pharmacogenomic associations in GLP-1RA-treated T2D cohorts. Therefore, *WFS1* variants are not established clinical predictors of GLP-1RA treatment response.

## 5 Metabolomic shifts induced by GLP-1RAs

Metabolite profiling may help characterize molecular pathways associated with GLP-1RA treatment and identify biomarkers of therapeutic response. Numerous studies have demonstrated treatment-induced metabolic shifts mainly in three metabolic domains including membrane-lipid remodeling, amino-acid and energy metabolism, and bile-acid/oxylipin signaling (Jendle et al., 2021; Du et al., 2022; Zhang L. et al., 2022; Alfadda et al., 2024). This section summarizes metabolomic alterations associated with GLP-1RA interventions in human studies.

### 5.1 Lipids

GLP-1RA treatment has been associated with remodeling of several lipid classes, including glycerophospholipids, sphingomyelins (SMs), ceramides (Cers), lysophosphatidylcholines (LysoPCs), phosphatidylethanolamines (PEs), lysophosphatidylethanolamines (LysoPEs), and phosphatidylcholines (PCs).

In 52 overweight or obese patients with newly diagnosed T2D, serum metabolomic profiling was performed before and after 12 weeks of Dulaglutide or Liraglutide treatment (Du et al., 2022). Partial least squares-discriminant analysis showed a clear separation between the metabolite profiles of patients with T2D and lean healthy controls, with T2D characterized predominantly by altered glycerophospholipid metabolism (Du et al., 2022). In the Dulaglutide group, PC (16:1/22:6), PE (16:0/0:0), and PS (O-20:0/14:0), PGP (LTE4/i-20:0) and LPC (10:0/0:0), which were elevated at baseline, decreased after treatment, whereas LysoPC (P-16:0 and P-18:0) and LysoPE (22:1) increased after 12 weeks of treatment. Comparable glycerophospholipid remodeling was observed with Liraglutide, although the individual molecular species differed between treatments (Du et al., 2022). These findings suggest that glycerophospholipid remodeling may represent a pharmacodynamic signature of GLP-1RA treatment, whereas changes in individual lysophospholipid species appear to be treatment- and cohort-specific.

In a separate study, 12 weeks of Exenatide treatment in patients with newly diagnosed T2D was associated with reductions in selected SM species including SM (d18:1/18:0), and SM (d18:1/18:1), as well as LPC (16:0) and LPE (18:0), which were elevated at baseline (Zhang L. et al., 2022). Changes in these lipids were associated with changes in BMI, fasting glucose, HbA1c, total cholesterol, LDL-C, triglycerides, and HOMA-IR (Zhang L. et al., 2022). These associations between lipid changes and improvements in BMI, glycaemia, HOMA-IR, and circulating lipids further support the clinical relevance of lipid remodeling. However, these associations remain observational and do not establish that changes in specific metabolites mediate clinical benefits.

Further pharmacometabolomic analysis of Liraglutide-treated patients with T2D identified significant changes in Cers, SMs, cholesterol esters, phosphatidylglycerols, and other glycerophospholipid species, including SM (40:2), LPC (18:0), SM (d36:1), SM (37:1), SM (d41:2), and PG (O-39:0) (Jendle et al., 2021). In a separate study of patients with obesity, 12 weeks of Liraglutide treatment increased DG [18:3(10,12,15)-OH (9)/0:0/8:0] and CDP-DG (a-17:0/20:4-2OH), among other altered endogenous metabolites (Alfadda et al., 2024). Liraglutide also altered oxylipins and arachidonic-acid-related pathways, indicating modulation of lipid mediators potentially involved in oxidative stress and inflammation (Alfadda et al., 2024).

Collectively, lipid-pathway changes are consistent with remodeling of membrane lipids, lipoprotein-associated lipids, and lipid mediators during GLP-1RA therapy. Reductions in selected SMs, Cers, and glycerophospholipids may be consistent with reduced lipotoxic stress and improved insulin sensitivity; however, these mechanisms cannot be inferred directly from circulating metabolite changes alone (Wu et al., 2022). In the Exenatide study, changes in several SMs, Cers, and phospholipid species were associated with improvements in HOMA-IR and other cardiometabolic measures (Zhang et al., 2022).

### 5.2 Amino acids

GLP-1RAs have also been associated with changes in amino acids (AAs), peptides and related metabolic pathways. In T2D, Jendle *et al.* reported that Liraglutide had a minor but statistically significant effect on selected AAs, including threonine, tryptophan, glycine, and valine compared with Glimepiride (Jendle et al., 2021).

In a 12-week Liraglutide intervention in patients with obesity, more pronounced shifts in peptide and amino-acid metabolism were observed. Beta-citryl-L-glutamic acid, creatine, leucine, glutamic acid, N-acetylaspartic acid and N-palmitoyl phenylalanine decreased after treatment, whereas cysteine, alanylvaline, Gly-Pro-Arg-Pro-Lys, and several N-acyl amino-acid derivatives increased (Alfadda et al., 2024). These findings support an effect of Liraglutide on amino-acid metabolism and related peptide pathways, alongside its broader effects on lipid and oxylipin metabolism.

In patients with recent-onset T2D, several amino acids, including glutamine, alanine, glycine, isoleucine, ornithine, phenylalanine, norleucine, lysine, acetylglutamine, 4-methylene-L-glutamine, isoglutamine, and histidine, differed from levels observed in healthy controls at baseline (Du et al., 2022). Following 12 weeks of treatment, the Dulaglutide group showed enrichment of pathways related to tricarboxylic acid cycle, β-alanine metabolism, glycolysis, histidine metabolism, and pyruvate metabolism, whereas the Liraglutide group showed enrichment of tryptophan metabolism (Du et al., 2022).

Exenatide treatment for 16 weeks in patients with T2D and endothelial injury also identified baseline amino-acid-related metabolites that differed between participants with greater versus lesser reductions in soluble thrombomodulin, an endothelial-injury marker. In particular, 4-hydroxyproline, pyroglutamic acid, and 5-amino-2-oxopentanoic acid were higher, whereas L-lysine and L-serine were lower in responders (Yang et al., 2021). However, these findings represent candidate baseline predictors of Exenatide-associated improvement in endothelial injury rather than validated markers of glycaemic or weight-loss response (Yang et al., 2021).

Overall, amino-acid findings are less consistent than lipid-pathway changes and appear to reflect treatment- and cohort-specific alterations in substrate utilization and energy metabolism. Most reported amino-acid changes were measured after treatment initiation and should therefore be interpreted as pharmacodynamic effects rather than predictive biomarkers.

### 5.3 Bile acids

Despite the metabolic benefits of GLP-1RAs, their use has been associated with an increased risk of gallbladder disease or biliary disease. A meta-analysis of 76 randomized trials found increased risks of composite gallbladder or biliary disease, cholelithiasis, and cholecystitis among GLP-1RA-treated participants, particularly at higher doses, with longer treatment duration (Ramirez-Mejia et al., 2025). GLP-1RA-associated gallbladder and biliary risk has been reported in randomized-trial meta-analysis, while the mechanisms remain incompletely defined. Potential mechanisms include changes in bile-acid metabolism, bile composition, and gallbladder motility.

In a 12-week study, Smits *et al.* showed that Liraglutide increased systemic concentrations of secondary bile acid deoxycholic acid in both fasting and postprandial states, as well as in feces. However, Liraglutide did not alter fasting gallbladder volume or gallbladder ejection fraction (Smits et al., 2016), suggesting that Liraglutide effects may be more apparent in bile acid metabolism than in gallbladder contractility.

Liraglutide’s effects on bile acids have also been explored in patients with obesity. In a 12-week intervention, post-treatment metabolomic profiling showed changes in several bile acid-related metabolites, in which trihydroxy-5β-cholanoic acid levels increased, whereas tetrahydroxy-5β-cholan-24-oic acid, urobilinogen, ursodeoxycholic acid and biotripyrrin-B decreased relative to pre-treatment levels (Alfadda et al., 2024). In contrast, only modest bile acids changes were observed in patients with T2D. Untargeted plasma metabolomics identified relatively small changes in bile-acid-associated metabolites, including taurolithocholic acid-3-O-sulfate, hyodeoxycholic acid and chenodeoxycholic acid-3-glucuronide, after Liraglutide treatment (Jendle et al., 2021). Similarly, in another T2D cohort, primary bile acid biosynthesis was identified in pathway analysis but had low pathway impact, suggesting limited bile-acid remodeling after short-term Liraglutide therapy (Benabdelkamel et al., 2024).

Overall, the metabolomic effects of GLP-1RAs appear to be dominated by remodeling of lipid-related pathways, including glycerophospholipids, sphingolipids, fatty acids, and oxylipins, whereas amino-acid and bile-acid findings have shown greater heterogeneity across studies. At present, most reported metabolic changes should be regarded as pharmacodynamic signatures of GLP-1RA treatment rather than validated predictors of clinical efficacy, since they were identified following treatment and have not been consistently associated with subsequent glycaemic, weight-loss, cardiovascular, or other clinical outcomes in independent cohorts.

## 6 GLP-1RAs and gut microbiota

Numerous findings indicate that GLP-1RA interventions modulate the gut microbiota and related metabolic pathways. Here, we summarize preclinical and clinical studies evaluating GLP-1RA-associated changes in gut-microbiota composition and their associated metabolic outcomes, as presented in Table 2.​

**TABLE 2:** Preclinical and clinical studies on GLP-1RA-induced changes in gut microbiota composition and their associated metabolic outcomes.

Model/Population	Intervention	Microbiota outcomes/Key numbers	Ref
Preclinical: Diet-induced obese mice	(Ex-4)_2_-Fc ± *Lactobacillus reuteri*	Beta diversity increased; *Lactobacillus reuteri*, *Akkermansia muciniphila*, *Bacteroides* spp. enriched; *Lactobacillus* spp. negatively correlated with ceramides	(Lin et al., 2020)
Preclinical: HFD-fed mice	Liraglutide	Alpha diversity (Chao1, observed species) increased; ratio decreased; *Akkermansia muciniphila* 1.9% → 33.2%, *Lactobacillus* & Parabacteroides increased; negative correlation with TC & LDL	(Zhao et al., 2022)
Preclinical: HFD and *ob*/*ob* mice	Liraglutide	Phylogenetic diversity increased; HFD mice*: Verrucomicrobia* 346%, *Proteobacteria* 8.7%, *Akkermansia* increased, *Helicobacter* decreased; *ob*/*ob* mice: *Thermotogae* 155%, *Proteobacteria* 2%, *Prevotella* decreased, *Johnsonella* increased	(Moreira et al., 2018)
Preclinical: Obese and diabetic rats	Liraglutide	Overall microbial diversity decreased (selective remodeling); *Akkermansia* & *Lactobacillus* increased; *Proteobacteria* decreased; inversely correlated with cholesterol & LDL	(Zhao et al., 2018)
Preclinical: Diet-induced obese mice	Liraglutide vs. GUB09-145	HFD reduced richness/diversity; Liraglutide: *Akkermansia muciniphila* & Oscillospiraceae increased, Lachnospiraceae decreased; modulated lipid/sulfur metabolism and two-component regulatory pathways	(Madsen et al., 2019)
Clinical: T2D, n = 19 Liraglutide, n = 18 metformin	Liraglutide vs. metformin	*Akkermansia* increased (Liraglutide); *Sutterella* increased (metformin); *Akkermansia* higher in short/intermediate diabetes duration	(Wang et al., 2018)
Clinical: T2D, n = 37	Liraglutide vs. metformin	Beta diversity significantly separated; *Akkermansia* increased (Liraglutide), *Sutterella* increased (metformin); *Akkermansia* & *Bacteroides* associated with disease duration; overall temporal stability	(Shang et al., 2021)
Clinical: Newly diagnosed T2D	Dulaglutide, once weekly	1 week: no change; 48 weeks: β-diversity increased; Chao1 & ACE decreased, Shannon & Simpson stable; *Firmicutes* decreased (p < 0.001), *Bacteroidota* increased (p = 0.005); *Ruminococcus* & *Blautia* decreased, *Bacteroides*/*Bifidobacterium*/*Lactobacillus* increased; *Akkermansia* early transient increase; functional pathways enriched (energy, lipid, carb metabolism, glycans, vitamins, cofactors)	(Liang et al., 2024)
Clinical: T2D, pilot n = 52	Liraglutide vs. Dulaglutide	Alpha diversity unchanged; beta diversity P = 0.004; *Bacteroides* higher in responders; 17 microbial features correlated with HbA1c reduction; microbiota predicts GLP-1RA response (AUROC 0.96)	(Tsai et al., 2021)

### 6.1 Preclinical evidence

In a preclinical model of diet-induced obesity, treatment with a long-acting experimental GLP-1RA based on exendin-4, (Ex-4)_2_-Fc, administered at a dose of 1.8 mg/kg once every 6 days, was shown to induce browning of white adipose tissue by modifying gut microbiota composition and ceramide metabolism, thereby enhancing thermogenesis and lipid metabolism. Mechanistically, (Ex-4)_2_-Fc remodeled gut microbiota composition, increasing β diversity and enriching beneficial taxa such as *L. reuteri* (*Lactobacillus reuteri*), *Akkermansia muciniphila*, and *Bacteroides* spp. Co-administration of *L. reuteri* with low-dose (Ex-4)_2_-Fc (0.45 mg/kg) further potentiated weight loss, fat browning, and glucose tolerance, highlighting a synergistic effect between GLP-1R activation and specific gut microbes. Lipidomic profiling revealed significant reductions in serum ceramides and sphingomyelins, lipid species linked to insulin resistance and obesity-related metabolic dysfunctions. These effects were accompanied by upregulation of Acer2, an enzyme promoting ceramide degradation, and correlation analyses showed that *Lactobacillus* species were negatively associated with ceramide levels, suggesting a microbiota-mediated mechanism for lipid regulation (Lin et al., 2020). These findings suggest that gut microbial alterations may represent a critical intermediate linking GLP-1 signaling with systemic energy balance.

Similarly, multiple studies have shown that Liraglutide exerts pronounced effects on gut microbial composition in preclinical models of obesity. In high-fat diet (HFD)-fed mice, Liraglutide treatment (0.2 mg/kg/day, subcutaneous for 12 weeks) reversed HFD-induced dysbiosis. The *Firmicutes*/*Bacteroidetes* (F/B) ratio, elevated by HFD, was reduced following Liraglutide treatment. Beneficial genera such as *Akkermansia*, *Lactobacillus*, and *Parabacteroides* were increased, with *A. muciniphila* increasing from 1.9% in HFD to 33.2% in the HL (HFD + Liraglutide) group (vs. 25.1% in normal control). α-diversity metrics (Chao1 and Observed species) were significantly higher in the HL group compared to HFD (*p* < 0.05), indicating restoration of microbial richness, while Shannon and Simpson indices also increased, reflecting greater diversity. Spearman’s correlation analysis showed that *Akkermansia* and other enriched taxa were negatively correlated with TC and LDL (*p* < 0.05), suggesting that Liraglutide-mediated improvements in dyslipidemia are partially microbiota-dependent. Overall, these results indicate that Liraglutide mitigates HFD-induced dyslipidemia by reducing weight gain, improving insulin sensitivity, and remodeling gut microbial communities, highlighting gut microbiota as a mediator of GLP-1RA metabolic benefits (Zhao et al., 2022).

Similarly, Liraglutide for 2 weeks in HFD-induced obese mice and *ob*/*ob* mice led to a significant reduction in body weight (approximately 7%) and in retroperitoneal, subcutaneous, and perigonadal fat mass. In HFD mice, adipocyte size decreased by 44%, and both glucose tolerance and insulin sensitivity were improved. Gut microbiota analysis revealed notable shifts in composition, in HFD mice, phylogenetic diversity increased, with a substantial expansion of the phylum *Verrucomicrobia* (+346%) and a decrease in *Proteobacteria* (−8.7%), accompanied by enrichment of *Akkermansia* and reduction of *Helicobacter*. In *ob*/*ob* mice, α-diversity also increased, with expansion of *Thermotogae* (+155%), a slight reduction of *Proteobacteria* (−2%), decreased *Prevotella*, and increased *Johnsonella*. Across both dietary models, *A. muciniphila* was consistently enriched following treatment (Moreira et al., 2018).

In a further preclinical study, Liraglutide was shown to reduce body weight and improve metabolic parameters in obese and diabetic rats, effects that were accompanied by significant modulation of gut microbiota composition. Microbiota analysis via 16S rRNA gene sequencing revealed a selective reduction in overall microbial diversity and a shift in the *Firmicutes*/*Bacteroidetes* ratio toward a lean-associated profile. Notably, Liraglutide consistently enriched beneficial genera, including *Akkermansia* and *Lactobacillus*, while reducing potentially pathogenic *Proteobacteria*. These microbial changes were inversely associated with serum cholesterol and LDL levels, supporting the concept that *Akkermansia* expansion underlies Liraglutide’s lipid-lowering effects (Zhao et al., 2018).

In a preclinical model of diet-induced obesity, Liraglutide, and GUB09-145, a dual GLP-1/GLP-2 agonist, were administered for 4 weeks, resulting in significant reductions in body weight and improvements in glucose homeostasis, with Liraglutide additionally decreasing fasting insulin levels. High-fat feeding markedly reduced gut microbial richness and diversity, whereas treatment with Liraglutide selectively reshaped the gut microbiota, promoting enrichment of *A. muciniphila* and members of Oscillospiraceae, while reducing specific Firmicutes species such as Lachnospiraceae. In comparison, the dual GLP-1/GLP-2 agonist GUB09-145 elicited similar microbial changes but showed a less pronounced effect on glycemic parameters. Functional metagenomic analysis revealed that Liraglutide modulates microbial lipid and sulfur metabolism, as well as two-component regulatory pathways, highlighting a mechanistic link between GLP-1R activation and microbiota function (Madsen et al., 2019).

Collectively, these preclinical studies provide mechanistic hypotheses linking GLP-1RA treatment, microbial remodeling, and changes in lipid metabolism, energy balance, and insulin sensitivity. However, these findings cannot be directly translated into humans due to physiology, baseline microbiota, diet, and drug exposure.

### 6.2 Clinical evidence

Preliminary clinical studies have examined the effects of GLP-1RAs on gut microbiota in people with T2D. In a human T2D cohort, treatment with Liraglutide (n = 19) induced distinct alterations in the gut microbiome compared to subjects maintained on metformin (n = 18) over a 42-day period. Liraglutide administration was associated with a significant enrichment of *Akkermansia*, a genus implicated in maintaining gut barrier integrity and metabolic homeostasis, whereas metformin treatment corresponded with a significant increase in *Sutterella*, a genus previously linked to metabolic dysregulation. These microbial shifts were consistent at both baseline and Day 42, independent of co-administration with a nutritional supplement (GSK2890457) or placebo, indicating that Liraglutide itself drives these specific changes. Interestingly, *Akkermansia* abundance was higher in patients with short or medium durations of diabetes compared with those with longer-standing disease, suggesting that the responsiveness of the gut microbiota to GLP-1RAs may decline as disease progresses (Wang et al., 2018).

In a randomized clinical study involving 37 patients with T2D, the effects of Liraglutide (n = 19) were compared with those of metformin (n = 18) on gut microbiota composition using 16S rRNA gene sequencing, with analyses performed at baseline and after 42 days of treatment (74 fecal samples in total). β-diversity analysis based on weighted UniFrac distances revealed a significant separation between the microbiota profiles of Liraglutide- and metformin-treated subjects (ANOSIM, *p* < 0.002), irrespective of the sampling time point. At the taxonomic level, Liraglutide treatment was associated with a significant increase in the relative abundance of the genus *Akkermansia* both at baseline (adjusted *p* = 0.048) and at Day 42 (adjusted *p* = 0.003), whereas metformin-treated subjects exhibited a significant enrichment of the genus *Sutterella* (baseline adjusted *p* = 0.038; Day 42 adjusted *p* = 0.041). At Day 42, *Akkermansia* abundance remained significantly higher in the Liraglutide group compared with the metformin group, even after adjustment for concomitant treatments (adjusted *p* < 0.05). No significant temporal changes in bacterial genera were observed within either treatment group between baseline and Day 42, indicating overall microbiome stability over time. In addition, the relative abundances of *Akkermansia* and *Bacteroides* were significantly associated with diabetes duration (adjusted *p* < 0.05), with higher *Akkermansia* levels detected in patients with short or intermediate disease duration compared with those with long-standing T2D (Shang et al., 2021). Taken together, these two clinical studies indicate that Liraglutide, consistently promotes *Akkermansia* expansion, reinforcing a treatment-specific effect on gut microbiota that may contribute to improved metabolic outcomes in T2D patients.

A longitudinal clinical study evaluated the effects of once-weekly Dulaglutide on gut microbiota in newly diagnosed T2D patients, with fecal samples collected at baseline, 1 week, and 48 weeks. Short-term exposure (1 week) did not alter α- or β-diversity, whereas long-term treatment (48 weeks) induced marked microbial reorganization (β-diversity, PCoA and ANOSIM, *p* < 0.05). Microbial richness (Chao1 and ACE) decreased, while Shannon and Simpson indices remained stable, indicating selective remodeling rather than global loss. Taxonomically, Dulaglutide reduced *Firmicutes* (*p* < 0.001) and increased *Bacteroidota* (*p* = 0.005). Genera associated with insulin resistance (*Ruminococcus*, *Blautia*) were reduced, while potentially beneficial genera (*Bacteroides*, *Bifidobacterium*, *Lactobacillus*) were enriched. *Akkermansia* showed an early transient increase, but this was not sustained over the treatment period. Correlation analyses linked fasting glucose and HbA1c positively to *Firmicutes*, *Ruminococcus*, and *Blautia*, and negatively to *Bacteroidota*, *Bacteroides*, and *Bifidobacterium*, while C-peptide was inversely correlated with *Ruminococcus*. Functional inference (PICRUSt2) indicated that only long-term Dulaglutide altered microbial metabolic pathways, enriching energy, lipid, carbohydrate metabolism, and biosynthesis of glycans, vitamins, and cofactors, suggesting that prolonged GLP-1R activation drives functional microbial adaptations contributing to metabolic benefits (Liang et al., 2024).

In a pilot study of 52 patients with T2D, treatment with Liraglutide (n = 22) or Dulaglutide (n = 30) for 12 weeks resulted in heterogeneous glycemic responses, with 34 patients classified as responders and 18 as non-responders. Although both drugs were analyzed together due to similar clinical indications and glycemic efficacy, Dulaglutide-treated patients were generally injection-naïve with less severe disease, whereas Liraglutide-treated patients more frequently had prior insulin therapy and more advanced diabetes. Across the cohort, β diversity of the gut microbiota differed significantly between responders and non-responders (*p* = 0.004), while α diversity was comparable. Responders exhibited a higher relative abundance of *Bacteroides* (28.5% vs. 16.9%, *p* = 0.003), and a set of 17 microbial features, including *Bacteroides* dorei, *Roseburia inulinivorans*, and *Lachnoclostridium* was associated with HbA1c reduction. Importantly, these microbial associations are attributable to GLP-1RA therapy as a class, rather than to a specific drug, as the study design did not allow for definitive separation of Liraglutide-versus Dulaglutide-specific effects. These findings suggest that GLP-1RA therapy reshapes the gut microbiota, potentially contributing to interindividual differences in glycemic response (Tsai et al., 2021).

Overall, current evidence supports that GLP-1RAs consistently remodel the gut microbiota, enriching beneficial taxa such as *Akkermansia* while reducing potentially harmful groups like *Proteobacteria*. These microbial changes parallel improvements in glucose and lipid metabolism, energy balance, and systemic inflammation. While causality remains to be fully established, microbiota remodeling emerges as a key mediator of GLP-1RA efficacy and a promising target for personalized metabolic interventions.

## 7 Integrating multi-omics and precision medicine in GLP-1RA therapy: future perspectives

### 7.1 T2D phenotypes and GLP-1RA response

T2D is increasingly recognized as a heterogeneous metabolic disorder, encompassing a spectrum of phenotypic subgroups that differ in pathophysiology, disease progression, and therapeutic response. Traditional approaches that treat T2D as a uniform entity may fail to capture clinically relevant variability, potentially limiting therapeutic efficacy and risk stratification (Nair et al., 2022). Advances in clustering analyses and machine learning have facilitated the identification of clinically different phenotypes, providing a framework for precision medicine interventions (Herder et al., 2024). Among these, five major subgroups have been consistently delineated: severe insulin-deficient diabetes (SIDD), severe insulin-resistant diabetes (SIRD), mild obesity-related diabetes (MOD), mild age-related diabetes (MARD), and classical diabetes. Classification is based on age at onset, BMI, HbA1c, and indices of insulin secretion and resistance, with each subgroup demonstrating distinct trajectories and complication profiles (Grandl et al., 2024; Sarria-Santamera et al., 2020). Moreover, metabolic heterogeneity is evident even in prediabetic states, where clustering analyses reveal subgroups with variable risks of progression to overt T2D and vascular complications (Neeland et al., 2024). Furthermore, assessments derived from OGTT combined with machine learning (ML) approaches have revealed substantial inter-individual variability in β-cell function and IR, underscoring the limitations of conventional classifications and the importance of incorporating continuous metabolic measures for risk stratification (Szekeres et al., 2024). Collectively, these findings emphasize the necessity for longitudinal phenotypic assessment to guide clinical decisions.

Phenotypic stratification also has direct implications for therapeutic interventions, particularly GLP-1RA therapy. Evidence indicates that patients with SIDD tend to achieve more pronounced reductions in HbA1c with GLP-1RA treatment, reflecting the drug’s insulinotropic effects in the context of β-cell deficiency. Conversely, SIRD patients may exhibit more modest glycemic improvements but derive additional benefits in weight reduction and other metabolic parameters, likely due to the modulation of insulin sensitivity and body composition (Caruso et al., 2025). Analyses involving 4,467 adults demonstrate that baseline characteristics, including age, sex, diabetes duration, BMI, and HbA1c significantly influence therapeutic response to GLP-1RA agents, highlighting the multifactorial determinants of efficacy (Heni et al., 2025). Large-scale precision medicine initiatives and frameworks, including consensus efforts by major diabetes associations (e.g., ADA/EASD), emphasize the potential for phenotype-guided and individualized treatment selection to improve glycemic and clinical outcomes compared with traditional one-size-fits-all approaches, although definitive trial evidence with tens of thousands of participants is still emerging (Chung et al., 2020). In older or frail adults, phenotypes characterized by sarcopenic-obesity require focused consideration, as these patients may achieve glycemic and weight benefits but remain at increased risk of adverse events if frailty is not accounted for (Sinclair and Abdelhafiz, 2025). These observations underscore the importance of integrating both metabolic and clinical phenotypes into individualized treatment planning. In summary, the integration of phenotypic stratification into clinical practice represents a critical step toward precision management of T2D. Tailoring GLP-1RA therapy to individual patient profiles, including β-cell function, IR, obesity, liver involvement, and frailty optimize therapeutic efficacy, mitigates adverse outcomes, and enables proactive management of diabetes-related complications. This approach exemplifies the transition from a one-size-fits-all strategy to a nuanced, phenotype-driven paradigm in T2D care, setting the stage for multi-omics and AI-driven innovations.

### 7.2 Multi-omics integration: pharmacogenomics, metabolomics and microbiomics

Interindividual variability in response to GLP-1RAs among individuals with T2D and obesity represents a major challenge to achieving optimal glycemic control and minimizing adverse effects. Treatment outcomes may arise through interconnected pathways in which host genetic variants, baseline metabolic state, gut-microbial composition, and clinical characteristics including HbA1c, BMI, and diabetes duration, influence GLP-1RA pharmacodynamics and collectively contribute to heterogeneous treatment response.

Variants in genes involved in GLP-1R signaling, including *GLP1R* have been associated with differences in HbA1c reduction following GLP-1RA therapy (Table 1). These variants may alter GLP-1R signaling and β-cell function, thereby modifying glycemic and weight responses (Li et al., 2020; Sathananthan et al., 2010; Lagou et al., 2023). In parallel, pharmacometabolomic profiling of individuals with T2D treated with GLP-1RAs, including Liraglutide, has revealed distinct circulating metabolic signatures, particularly in lipid and sphingolipid pathways, that differ from other glucose-lowering therapies (Jendle et al., 2021). These signatures may reflect both baseline determinants and downstream consequences of drug response.

In a phase II obesity-trial substudy, integrated proteomic, metabolomic, and lipidomic profiling of 25 bofanglutide-treated participants identified glycogenin 1 (*GYG1*) as a candidate biomarker and central network hub associated with both baseline prediction and longitudinal monitoring of GLP-1RA associated weight-loss response (Li et al., 2026). However, bofanglutide remains an investigational GLP-1RA undergoing clinical evaluation (Liu et al., 2026). Together, these findings provide a rationale for developing integrated multi-omics predictive frameworks for personalized GLP-1RA therapy.

Furthermore, weight loss induced by GLP-1RAs is associated with reductions not only in fat mass but also in lean body mass. Variability in the extent of lean-mass change across different GLP-1RAs underscores the importance of considering body composition adaptations when evaluating metabolic outcomes, as preservation of lean mass may influence metabolic homeostasis and therapeutic efficacy (Karakasis et al., 2025). Furthermore, selected GLP-1RAs improve lipid metabolism and adipose tissue function, as evidenced by reductions in serum lipids, visceral fat, adipose inflammation, and enhanced adipocyte metabolism, while also mitigating muscle catabolism through enhanced myocyte metabolism and reduced inflammatory signaling (Szekeres et al., 2024). These observations emphasize the need to consider phenotypic characteristics, including body composition, alongside molecular predictors to improve patient stratification.

Beyond host metabolic and phenotypic factors, the gut microbiota may provide a mechanistic interface between the baseline metabolic state and GLP-1RA response. Microbial communities generate metabolites, including short-chain fatty acids and secondary bile acids, that can influence intestinal barrier integrity, immune signaling, adipose-tissue metabolism, and endogenous incretin biology (Visekruna and Luu, 2021; Mann et al., 2024). Conversely, GLP-1RA therapy may indirectly alter gut-microbial composition and function through changes in food intake, body weight, gastrointestinal transit, and bile-acid metabolism (Kamath et al., 2026). Thus, the relationship is likely bidirectional, in which the baseline microbial and metabolic features may influence treatment response, whereas GLP-1RA treatment may subsequently remodel microbial composition and metabolite production.

Preclinical evidence supports this proposed microbiota-metabolite-host pathway. In obese mice, treatment with the long-acting GLP-1RA (Ex-4)_2_-Fc was associated with enrichment of *A. muciniphila* and increased microbiota-derived inosine. Inosine activated adenosine A2A signaling in macrophages, reduced adipocyte leptin expression, reversed hypothalamic leptin resistance, and improved obesity-related outcomes (Dong et al., 2025). This model provides a plausible causal pathway through which GLP-1RA-associated microbial remodeling may contribute to metabolic benefit, however, validation in GLP-1RA-treated human populations is required.

Conceptual frameworks for precision GLP-1RA therapy aim to integrate multi-omics layers with clinical variables to stratify patients according to both expected efficacy and risk of adverse events (Tan et al., 2025). Such integrative approaches may support individualized treatment strategies by combining molecular, metabolic, microbial and phenotypic information to optimize efficacy while minimizing treatment-related risks.

### 7.3 AI-driven predictive modeling and clinical implementation

Artificial intelligence (AI) approaches, including ML and neural-network methods are emerging as transformative tools which may help in interpreting complex clinical and multi-omics datasets to support precision-guided GLP-1RA therapy (Zou et al., 2023; Corrao et al., 2025). In principle, ML can model nonlinear and high-dimensional interactions among demographic and clinical characteristics, prior medication exposure, genetic variants, proteomic or metabolomic markers, and gut-microbial features (Tan et al., 2025; O'Sullivan et al., 2025). Such approaches may facilitate patient stratification, identification of responders and non-responders, and prediction of metabolic outcomes (Ronn et al., 2024). A systems-level framework integrating baseline determinants, longitudinal multi-omics changes, and AI-driven prediction is summarized in Figure 2.

**FIGURE 2:**
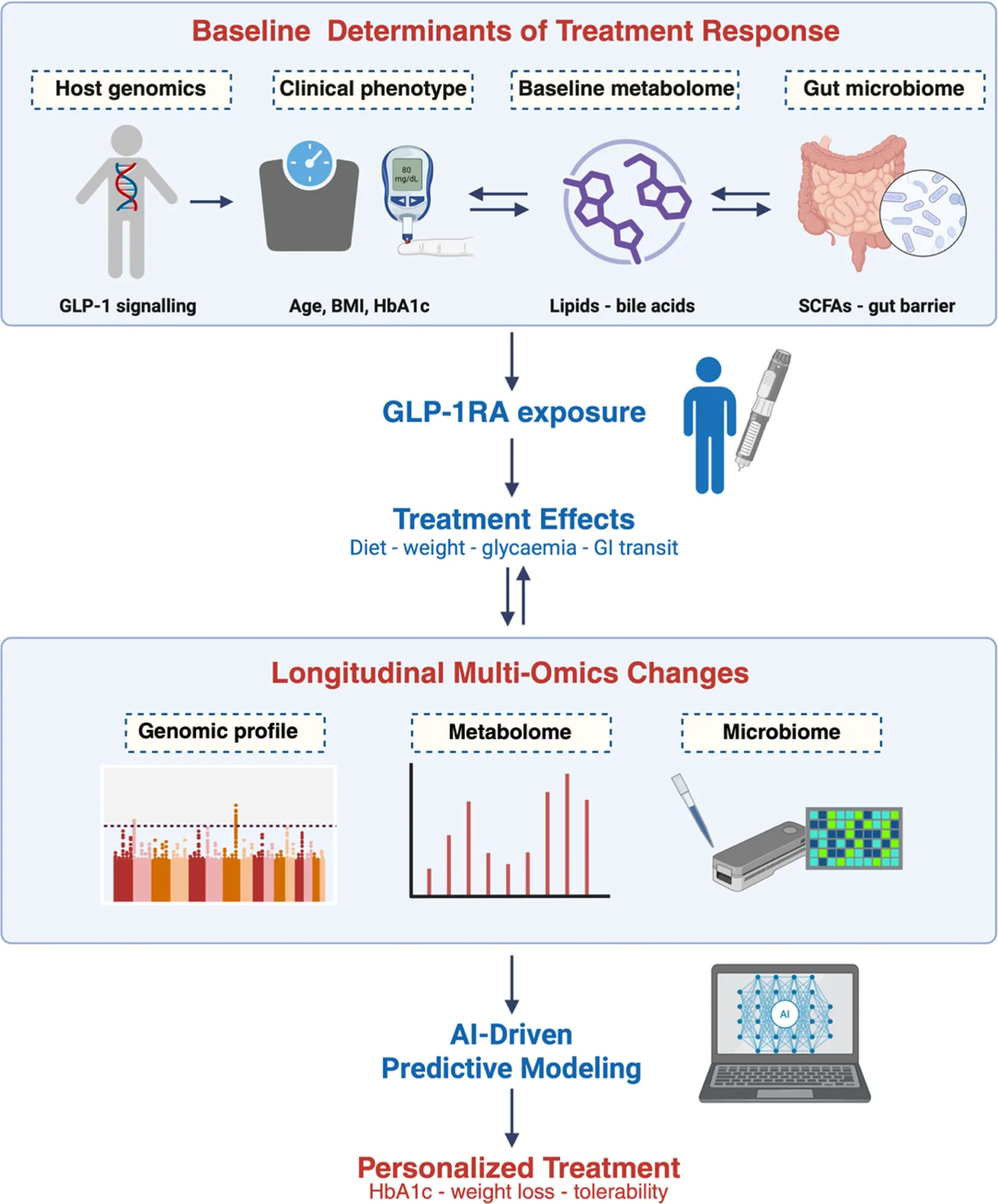
Systems-level framework for precision GLP-1RA therapy in T2D and obesity. Host genomics, clinical phenotype, baseline metabolome, and gut microbiome represent interconnected baseline determinants of GLP-1RA treatment response. GLP-1RA exposure may induce changes in diet, body weight, glycaemia, and gastrointestinal (GI) transit, which may contribute to longitudinal metabolomic and microbiome alterations. Integrative predictive modelling can combine baseline and longitudinal clinical and multi-omics data to support personalized treatment according to expected glycaemic response, weight loss, and tolerability. SCFAs, short-chain fatty acids; GLP-1RA, glucagon-like peptide-1 receptor agonist. Created by BioRender.com.

Direct ML applications for GLP-1RA response prediction are emerging but remain limited. In an exploratory analysis of the Diastrat cohort, Villikudathil *et al.* evaluated clinical, genomic, and proteomic correlates of GLP-1RA response in individuals with T2D (Villikudathil et al., 2024). Five ML algorithms were trained separately using selected clinical or proteomic features to distinguish GLP-1RA responders from non-responders. The best proteomic Naïve Bayes model achieved a classification accuracy of 87.72% ± 3.65%, an Area Under Curve (AUC) of 0.70, and a Matthews correlation coefficient of 0.53. While the best clinical Naïve Bayes model achieved an AUC of 0.71 and classification accuracy of 91.23% ± 3.46% (Villikudathil et al., 2024). Although the study identified clinical, proteomic, and genomic correlates of response, the predictive models were limited to separate clinical-only and proteomic-only analyses, since genotype data were limited.

In a larger real-world study, Zhu *et al.* developed and validated supervised ML models to predict treatment responsiveness using electronic health record data to predict glycemic responsiveness before initiation of GLP-1-pathway medications, including GLP-1RAs and dipeptidyl peptidase-4 inhibitors (DPP-4i) (Zhu et al., 2024). Higher baseline HbA1c, higher BMI, older age, and shorter T2D duration were associated with better treatment responsiveness. In contrast, Sulfonylurea or thiazolidinedione use, Asian ancestry, heart failure, retinopathy, higher blood chloride, and longer T2D duration contributed negatively to model’s predicted value of treatment effectiveness (Zhu et al., 2024). However, this study included both GLP-1RAs and DPP-4i, therefore, it findings cannot be considered exclusively predictive of GLP-1RA response.

Complementing these ML studies, Tsai *et al.* developed a clinical-adjusted, microbiome-based linear regression model to predict HbA1c reduction after GLP-1RA therapy in patients with T2D (Tsai et al., 2021). Baseline enrichment of *Bacteroides dorei* and *Lachnoclostridium* sp. was associated with greater HbA1c reduction, whereas higher baseline abundance of *Mitsuokella multacida* was associated with a poorer glycemic response (Tsai et al., 2021). Although Tsai *et al.* identified gut-microbial signatures associated with glycemic response to GLP-1RA therapy using supervised ML approach, ML- and deep-learning-based models that integrate gut microbiome data with clinical and multi-omics features to predict GLP-1RA responsiveness remain largely unexplored.

Based on the above-mentioned studies, ML and related predictive-modelling approaches have shown promise for identifying biomarkers associated with GLP-1RA therapeutic response (Ahmed et al., 2025). However, currently available models are largely based on clinical data or individual molecular data layers, rather than integrated multi-omics datasets. Prospective validation of multimodal models combining clinical, genomic, proteomic, metabolomic, and microbiome data features is warranted to improve GLP-1RA response prediction.

Integration of multiple omics layers may generate mechanistic insights beyond conventional clinical parameters and support more individualized treatment stratification (O'Sullivan et al., 2025; Zhu et al., 2021). However, barriers to routine clinical implementation remain, including standardization of multi-omics data collection, harmonization of analytical pipelines, model interpretability, and external validation across diverse populations. Integration into practice also requires regulatory and ethical oversight, as well as clinician-friendly decision-support systems (Zou et al., 2023; O'Sullivan et al., 2025). Nevertheless, AI-driven predictive modeling has considerable potential to advance precision GLP-1RA therapy by bridging mechanistic understanding with real-world clinical decision-making and paving the way for personalized management of T2D and obesity.

### 7.4 Limitations and future directions

Despite the promise of precision GLP-1RA therapy, the current evidence base remains limited by small sample sizes, short treatment durations, and heterogeneous study populations. Differences in ancestry, T2D severity, obesity, baseline HbA1c and BMI, prior medications, GLP-1RA agent, dose, and definitions of treatment response limit cross-study comparisons and may contribute to inconsistent findings. Additionally, pharmacogenomic and metabolomic studies have predominantly evaluated glycaemic and weight-loss responses or post-treatment metabolic changes, rather than the prospective prediction of adverse events during long-term GLP-1RA use. Consequently, the potential of omics approaches to identify individuals at increased risk of GLP-1RA-associated adverse events warrants further investigation. To date, no genomic, metabolomic, proteomic, or microbiome biomarkers have been clinically validated to predict GLP-1RA intolerance or serious adverse events. Therefore, treatment decisions should currently rely on established clinical risk factors and routine safety monitoring.

Moreover, integrated multi-omics studies may help explain interindividual variability in GLP-1RA treatment response. For example, an integrated proteomic, metabolomic, and lipidomic analysis identified GYG1 as a candidate biomarker associated with baseline prediction and longitudinal monitoring of weight-loss response to the investigational GLP-1RA Bofanglutide (Li et al., 2026). However, whether such omics markers improve prediction beyond standard clinical assessment has not been established. Future precision-medicine studies should integrate baseline genomic, metabolomic, proteomic, and microbiome profiles with clinical risk factors and prospectively adjudicated safety outcomes to identify patients at risk of adverse events.
